# Critical signs and symptoms for self-assessment in the immediate postnatal period: an international Systematic Scoping Review and Delphi consensus study

**DOI:** 10.1186/s12884-025-07472-9

**Published:** 2025-03-28

**Authors:** Teesta Dey, Nada Bassiony, Angela Hancock, Lenka Benova, Matthews Mathai, Etienne Vincent Langlois, Sam Ononge, Tina Lavender, Andrew Weeks

**Affiliations:** 1 University of Liverpool, Liverpool, United Kingdom; 2 University Hospitals of North Midlands NHS Trust, Stoke, United Kingdom; 3 The Institute of Tropical Medicine (ITM), Antwerp, Belgium; 4 Liverpool School of Tropical Medicine (LSTM), Liverpool, United Kingdom; 5 Partnership for Maternal, Newborn and Child Health (PMNCH), World Health Organization (WHO), Geneva, Switzerland; 6 University of Makerere, Kampala, Uganda

## Abstract

**Background:**

Every two minutes a woman dies from complications of pregnancy and childbirth. Most maternal deaths occur within the first 24 hours following birth, highlighting the importance of immediate postnatal care (iPNC). Self-care strategies are increasingly being employed to promote women-centred, continuous care provision. Despite international calls for development of strategies promoting self-care, none have been developed for self-monitoring in the immediate postnatal period. Fundamental to the development of a self-monitoring strategy, is an understanding of which signs and symptoms are predictive of maternal morbidity and mortality and can be easily assessed by mothers and birth companions, in health facilities, without the need for equipment. The objective of this study was to develop and achieve international consensus on the key signs and symptoms.

**Methods:**

A multi-step approach involving a systematic scoping review, two- round Delphi Survey, and expert consensus was employed to identify key signs and symptoms that can be self- assessed and predict morbidity and mortality in the immediate postnatal period.

**Results:**

A comprehensive list of 351 key signs and symptoms was identified from 44 clinical practice guidelines. Subsequently, 134 signs and symptoms were reviewed by Delphi respondents and international expert consensus was achieved for 19 key signs and symptoms across seven condition categories. The signs that were considered both important and able to be self-assessed by mothers and birth companions in the first 24 hours following birth included change in consciousness, seizure, severe headache, persistent visual impairment, urinary incontinence, chest pain, shortness of breath, severe pallor, fast heartbeat, rejection of baby, suicidal/infanticidal, fever, heavy blood loss, soft flabby uterus, unable to urinate easily, foul smelling discharge, rigors, syncope/dizziness, abnormal coloured urine.

**Conclusion:**

This study identified key signs and symptoms which can be easily assessed by mothers and birth companions in the immediate postnatal period to identify those most at risk of morbidity and mortality. Further work is needed to validate this screening tool, and adapt it regionally and nationally.

## Background

According to the latest United Nations (UN) estimates, a woman dies from complications of pregnancy and childbirth every two minutes [1]. Most maternal mortality occurs within the first 24 hours and a focus on immediate postnatal care (iPNC) is therefore important [2, 3]. Despite its lifesaving value, postnatal care is critically neglected. One in five mothers and babies do not have access to life-saving postnatal care interventions [4]. Furthermore, the COVID-19 pandemic resulted in increasing fragmentation and disruption of essential reproductive health services including postnatal care. There is an urgent need for new and innovative strategies for postnatal care to ensure the accelerated reduction in global maternal deaths needed to achieve the Sustainable Development Goal Target 3.1 by 2030 [1].

Recent global crises have highlighted self-care strategies as effective mechanisms to ensure continued provision of services, promote women centred care and achieve universal health coverage. As such the World Health Organization (WHO) has made a call for strategies and interventions that promote self-care [5]. Yet within the existing list of self-care interventions, there is a lack of strategies for self-monitoring in the immediate postnatal period by mothers, supported by their birth companions, in health facilities [6].

Developing self-monitoring strategies requires an understanding of the key signs and symptoms and whether these could be easily assessed by mothers and birth companions in the immediate postnatal period. To date, there is no existing research nor consensus on this. The objective of this study was to achieve international consensus on a list of key immediate postnatal signs and symptoms that are predictive of maternal morbidity and which mothers, or their birth companions could self-assess in health facilities during the immediate postnatal period, without the need for additional equipment.

## Methods

A multi-step approach was utilised for this process including systematic scoping review, expert review and Delphi survey (Table 1).

**Table 1:** Process for establishing content of the Immediate Postnatal Women’s Assessment (ImPoWA) tool

Step	Aims	Participants	Methods
1	Systematic scoping literature review to generate a comprehensive list of signs and symptoms that clinical practice guidelines suggest should be assessed in the immediate postnatal period	N/A	Systematic scoping literature review
2	Review of systematic scoping review findings to agree on the content validity of key signs and symptoms for inclusion in Delphi Survey	Expert Committee	Group Discussion
3	Rating of the importance of signs and symptoms, and suggestion of any new additions	Delphi respondents (identified through snowball sampling)	Delphi Survey
4	Repeat of step 3 with the refined list	Invitations to same Delphi respondents as in step 3 above	Delphi Survey
5	Review of unresolved signs and symptoms, and achieving final consensus on tool content.	Expert Committee	Group Discussion

### Step 1: systematic scoping review

A systematic scoping review was undertaken to identify a comprehensive list of signs and symptoms that international clinical practice guidelines (CPG’s) suggest should be assessed within the immediate postnatal period. The review question was broad and as such PROSPERO advised the authors to conduct a scoping review. That said, the authors were committed to conduct the review rigorously and adopt a systematic approach. As such, the review was also conducted in accordance with PRISMA guidelines [7].

The most recent versions of CPGs, available in English and published between January 2010 and June 2020, were included. Guidelines were excluded that were not specific to postnatal mothers or did not specify signs or symptoms to be assessed during the postnatal period. Primary and secondary research studies, conference abstracts, locally created CPGs, or those focusing on COVID-19 were excluded.

A three-step search strategy was used (Appendix 1). Firstly, a comprehensive search of 15 published and three unpublished databases electronic databases was conducted. Secondly, a search of maternal health professional organisations and societies websites was conducted. Thirdly, the reference lists of selected guidelines were reviewed to identify additional CPGs.

Title and abstract screening was conducted by one reviewer (NB). Full text screening was conducted by two reviewers (TD, NB), with a third reviewer (ADW) available to discuss any disagreements. Data was extracted by TD and NB in duplicate to a pre-created data extraction form. Signs and symptoms were transcribed literally before being organised into categories according to the clinical condition they related to, based on clinical opinion. Any duplicated signs or symptoms were removed at this stage.

The quality of included CPGs was assessed by two reviewers (TD, NB) using the AGREE II tool with a third reviewer (ADW) consulted to discuss discrepancies [8]. In line with previous literature, a score of >60% for each domain was considered sufficient [8–10]. Finally, two reviewers (TD, NB) provided an overall assessment of each guideline, using the following parameters:

1. ‘Recommended’ was assigned if most domains (four or more) scored above 60%.
2. ‘Recommended with modifications’ was assigned if most domains (four or more) scored between 30–60%.
3. ‘Not recommended’ was assigned to any guidelines where most (four of more) of the domains scored below 30%.

Guidelines listed as ‘recommended’ or ‘recommended with modifications’ were selected for inclusion. Those guidelines listed as not recommended were excluded.

### Step 2: Expert Committee

A committee of nine experts was purposively selected from members of international and national groups focused on optimising postnatal care provision and uptake (Appendix 2).

The Expert Committee reviewed the systematic scoping review findings to assess content validity. Discussions focused on three questions:

- How likely are these signs and symptoms to occur in the first 24 hours following birth?
- How essential or likely is the sign or symptom to predict maternal morbidity and morbidity within 24 hours of birth?
- Can the sign or symptom be easily assessed by a mother or her birth companion without extensive training?

The Expert Committee were invited to suggest any additional signs and symptoms they deemed pertinent for inclusion.

### Step 3 and 4: Delphi Survey

A two-round anonymised electronic Delphi Survey was designed on Joint Information Systems Committee (JISC) software [11].

E-mail invitations, including a link to the Delphi Survey, were sent to relevant contacts of the research team to participate in the study. Invitees were encouraged to share the survey in their network resulting in snowball recruitment. The survey was also advertised through social media to gain a global response. Newly identified participants were sent personal e-mail invitations to participate in the study including a link to the survey.

There are no clear guidelines on sample size calculations required for a Delphi Survey [12, 13]. Previous models have suggested that a minimum of five in each area of expertise would be sufficient to be provide content validity and varied input to produce meaningful and generalisable results [12, 13]. Initial stakeholder mapping identified four key stakeholder groups to be included (Clinicians, Academics/Researchers, Public Health officers and programmers, Women’s Representatives). The researchers aimed towards a minimum sample size of 50 participants to account for potential attrition in Round 2 and likelihood of respondents crossing stakeholder groups.

During the Delphi Survey, participants were asked to rate the importance and possibility for mothers and birth companions to assess each of the signs and symptoms. Participants were asked to rate between 1 and 7 on a Likert scale with 1–2 being ‘not important’, 3–5 being ‘important but not critical’ and 6–7 being ‘critically important’. Participants had the option to select “unable to comment”.

In round 1, a free-text option enabled participants to suggest additional signs and symptoms that were not already listed.

Consensus for the Delphi Survey was defined *a priori*based on the limits used to develop the core outcome sets [14]. In this study, for an item to have achieved full consensus and be termed ‘critically important’, at least 75% of participants needed to score the item as ‘critically important’ with <15% of participants scoring the item as ‘not important’. Items that did not achieve consensus and were scored ‘not important’ required at least 75% of participants to score it as such and <15% to score the item as ‘critically important’. Any items not meeting either category would have achieved some consensus and be termed “somewhat important”.

A sensitivity analysis was conducted for signs and symptoms graded as “somewhat important”. Signs and symptoms with similar phrasings were arranged under a specific sign/symptom category irrespective of condition. The highest score each respondent attributed to any of the signs and symptoms within that category was recorded. Each sign and symptom was graded using the Likert scale, as above.

### First round of the Delphi Survey

The first Delphi Survey was open for four weeks. Two reminder emails were sent to non-responders across the survey period.

During the first round of the Delphi Survey, no signs or symptoms could be excluded. An additional 10 signs and symptoms were suggested by the respondents and added, resulting in a new questionnaire with 144 signs and symptoms for the second round of the Delphi Survey.

### Second round of the Delphi Survey

Responders to the first Delphi survey were also invited to participate in the second round. No new participants were invited at this stage and non-responders from round 1 were not invited to participate in round 2.

In Round 2, the revised questionnaire containing the 144 signs and symptoms was emailed directly to the Round 1 Delphi respondents. Within the survey, participants were provided with the results from round 1 (percentage of participants rating each sign or symptom as critically important), based on Delphi methodology. This enabled those receiving the survey to reflect on existing responses before completing round 2 [15].

The Delphi Survey closed four weeks after the start of round 2 and weekly reminder emails were sent until closure of the questionnaire.

### Step 5: Consultation meeting

The Expert Committee met to discuss the results of round 1 and 2 of the Delphi Survey. Prior to the meeting, the committee were provided with the collated results from both rounds of the survey. The committee members were asked to prioritise the list of key signs and symptoms deemed most critical (>75% or above). Duplicates, those considered difficult for the mothers and birth companions to assess, and those occurring over 24 hours after birth were removed from the list. Next the list of signs and symptoms deemed ‘somewhat critical’ were reviewed. Duplicates, and those considered difficult for the mothers and birth companions to assess, were removed from the list. Finally, the committee reviewed all results to reach a final consensus on the key signs and symptoms that could be self-assessed by mothers, with the support of their birth companions.

Ethics approval for this study was gained through University of Liverpool Ethical Review Committee (Ref: 9743).

## Results

### Step 1: Systematic scoping review

A total of 20,734 articles relevant to iPNC were identified (Appendix 3) were screened. Forty-four CPGs were identified, which met eligibility criteria, and were included in the review (Appendix 4). Of these guidelines, 13 were intended for an international audience, 30 were specifically for high income countries and six were created for low- and middle-income country (LMIC) settings. Only 11 guidelines were specifically for the postnatal period.

A total of 351 maternal signs and symptoms, across 12 condition categories pertaining to the immediate postnatal period, were identified.

### Step 2: Expert Committee

The Expert Committee reviewed the initial list of 351 signs and symptoms for content validity. One duplicate was removed, and 232 signs and symptoms were excluded. One hundred and seven were deemed unlikely to occur within the first 24 hours of birth; 45 were not considered essential for predicting maternal morbidity and mortality; and 79 were considered unsuitable for assessment by a mother or her birth companion, without additional equipment. Alternative phrasing was proposed for six signs and symptoms that had previously been excluded and as such were re-added. An additional nine signs/symptoms were suggested. Discussions resulted in the creation of a list of 134 signs and symptoms, arranged in seven condition categories, to be reviewed during the first round of the Delphi Survey (Appendix 5).

### Steps 3 and 4: Delphi Survey

One hundred and thirteen respondents, from 10 countries, completed round one of the Delphi Survey. Fifty-nine of these respondents, from nine countries, subsequently completed round two (52%) Most of the round two respondents (94%) were practising health care workers (Table 2). Most respondents had been working in clinical practice for at least 5 years. There were 51% of respondents from high-income-settings, 37% from lower-middle-income settings and 12% from low-income settings.

**Table 2:** Demographics of Delphi respondents

		**Round 1** (*n*=113)		**Round 2** (*n*=59)	
		Number	%	Number	%
**Role**	Clinicians	101	89	55	94
	Academics/researchers	6	6	2	3
	Public Health officers and programmers	5	4	2	3
	Women’s representatives	1	1	-	-
**Length of Duty**	>10 years	61	54	31	53
	5–10 years	26	23	16	27
	2–4 years	15	13	6	10
	1 year or less	11	9	6	10
**Country**	UK	40	36	28	48
	Nigeria	22	19	7	12
	Uganda	16	14	7	12
	Kenya	14	12	6	10
	Nepal	11	9	4	7
	Tanzania	2	2	2	3
	USA	2	2	2	3
	India	4	4	2	3
	Ghana	1	1	1	2
	Australia	1	1	-	-

### Step 5: Expert Committee

**Fig. 1:**
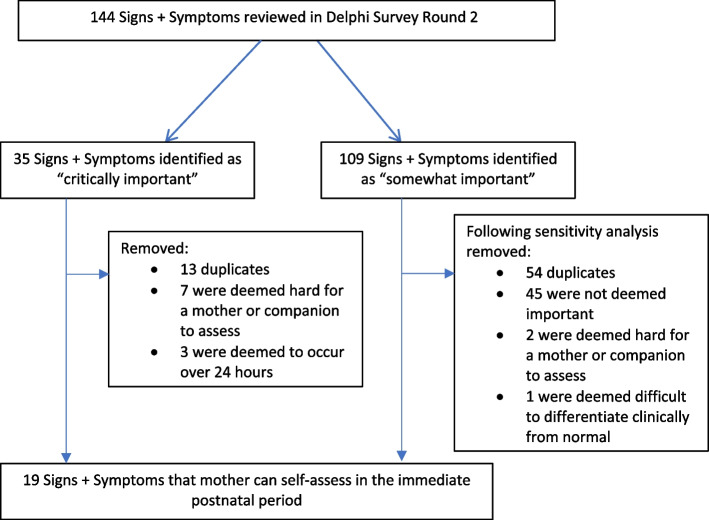
Data flow chart for Expert Committee discussion

When reviewing the results of each round of the Delphi Survey, the Expert Committee highlighted the value of a broad list of signs and symptoms, not limited to specific conditions occurring in the postnatal period, given that many signs and symptoms span multiple conditions. Of the 144 signs and symptoms reviewed in round 2 of the Delphi survey, 35 (24%) were considered ‘critically important’; 109 (76%) ‘somewhat important’; and none were deemed unimportant or excluded (Fig. 1).

### ‘Critically important’ signs and symptoms

Of the 35 most critically important signs, the Expert Committee excluded 23 of them. Thirteen were duplicates; 10 were deemed difficult for a mother and birth companion to assess without training and equipment or would occur after 24 hours following birth. Twelve were selected for inclusion in the list of key signs and symptoms. These included, “change in consciousness, seizure, severe headache, persistent visual impairment, urinary incontinence, chest pain, shortness of breath, severe pallor, fast heartbeat, rejection of baby, suicidal/infanticidal, fever”.

### ‘Somewhat important’ signs and symptoms

For the 109 somewhat important signs and symptoms, a sensitivity analysis was conducted. Ten signs and symptoms categories contained at least one sign or symptom that was scored as “critically important” by more than 75% of respondents. These were dizziness, amount of blood loss, foul smelling discharge, hallucinations/delusions, inability to pass urine, depression, rigors, lethargy, coloured urine and soft flabby uterus. Fifty-four signs and symptoms were listed within the ten sign and symptoms categories and excluded as duplicates. Forty-five signs and symptoms were not housed within the ten important signs and symptoms categories and excluded as deemed not important.

The Expert Committee advised renaming two of the signs and symptoms for clarity. Coloured urine was renamed “abnormally coloured urine”, and amount of blood loss was renamed “heavy blood loss”.

Three signs and symptoms; “lethargy”, “hallucinations and delusions” and “depression” were excluded as the Expert Committee considered them to be difficult for a mother and birth companion to assess.

A final list of 19 signs and symptoms that were important and possible to be assessed in the immediate postnatal period were selected as below:

**Table:** 

Change in consciousness
Seizure
Severe Headache
Persistent visual impairment
Urinary incontinence
Chest pain
Shortness of breath
Severe pallor
Fast heartbeat
Rejection of baby
Suicidal/infanticidal
Fever
Syncope/dizziness
Heavy blood loss
Foul smelling discharge
Unable to urinate easily
Rigors
Abnormal coloured urine
Soft flabby uterus

## Discussion

### Main findings

This study achieved its aims of developing consensus on the key signs and symptoms, predictive of maternal morbidity and mortality in the immediate postnatal period (first 24 hours following birth), that could be self-assessed by mothers supported by their birth companions. A list of 19 key signs and symptoms, spanning seven condition categories (postpartum haemorrhage (PPH), genital tract sepsis, cardiovascular disease, preeclampsia/eclampsia, urinary dysfunction, anaemia, postpartum psychosis), was developed. The research team believe this to be the first evidence-based self-care strategy for use in the immediate postnatal period to be developed.

### Strengths and limitations

The study has several strengths. First, a mixed methods approach was designed . A three-step systematic scoping review identified an expansive list of signs and symptoms from CPGs. Quality assessment was completed using the AGREE II tool to ensure only signs and symptoms from reputable guidelines were included [16]. The Delphi method enabled the involvement of a diverse range of lay and professional stakeholders from geographically distant regions. The use of snowball recruitment using the social media platforms was a useful method of recruitment. Over 90% of participants had at least two years of professional experience. Although the study was led by a UK based research team, there was representation from higher income settings and lower to middle income settings within the Expert Committee and Delphi respondents to enhance the generalisability of the results. The Delphi process also enabled participants to consider the views of others and develop their own opinions. Discussion and debate by the Expert Committee led to further refinement and agreement of the final tool.

There are several limitations to consider. First, although there was a large participation in the survey, representation from each stakeholder group was not evenly distributed with 93% of respondents being health workers in round 2 and there was only one patient representative in round 1. It is likely that some participants belonged to more than one stakeholder group, but data are not available to explore this further. Secondly, no signs and symptoms were deemed “not important” during the Delphi process and could be removed. This is unsurprising as all signs and symptoms were retrieved from international recommendations and as such will all be somewhat important at the very least. The limits for consensus were developed *a priori*and in line with existing Delphi studies [14, 17]. It might however have been prudent to have developed a limit for the ‘somewhat important’ category too or utilised an alternate method for rating such as ranking of outcomes.

### Interpretation

The four conditions with the highest number of recommendations associated were postpartum haemorrhage, pre-eclampsia/eclampsia, genital tract sepsis and anaemia. Global findings indicate that PPH, Pre-eclampsia/eclampsia and genital tract sepsis account for more than half of maternal deaths worldwide [18]. Additionally, anaemia is widely regarded as a risk factor for worsening outcomes in those experiencing PPH [18]. Given the high morbidity and mortality associated with these conditions, it would seem logical that more guidelines are available that focus on them. However, most guidelines are specifically for high income settings. This is problematic given that the highest maternal morbidity and mortality occurs in LMIC’s. Additionally, there were few country-specific recommendations which are critical to implementing guidelines into clinical practice. Absence of national guidelines and local protocols in maternal health in LMIC’s has been highlighted as a key barrier preventing implementation of high-quality care [19]. Contextualised guidelines, to promote and support consistent delivery of high-quality care in these settings, are urgently needed.

There were no CPGs focussing solely on the immediate postnatal period. Of the 44 guidelines included in the review, only 25% (11 guidelines) were specified for the postnatal period. Most guidelines covered the antenatal, intrapartum and postnatal period. This was highlighted in a previous systematic review, with only six international guidelines focussing specifically on postnatal care [20]. Over the past decade, there has been a move to promote continuity of care, through integration of services. The benefits, and improved health outcomes from this approach, are well documented [19, 21]. However, in addition to integration, there is a need to ensure renewed priority to poorly covered services such as iPNC where the morbidity and mortality is greatest [21, 22]. Development of specific clinical guidelines on postnatal care would provide the much-needed focus on key health issues, guiding health care providers, programme officers and policy makers in providing comprehensive, high-quality care.

The quality of CPGs reviewed varied greatly with a lack of detail and transparency of the development processes by the guideline developers. These findings are consistent with other quality assessments of clinical practice guidelines in maternal care [20, 23, 24]. There is a need for guideline development processes to be made explicit, to ensure the content is evidence based and enable practitioners to make informed decisions about whether to adopt the guidance.

There is a paucity of literature on danger signs and symptoms specifically within the first 24 hours of birth. For example, the 2022 WHO postnatal care guidelines and the Ugandan Clinical Guidelines only mention danger signs and symptoms for ongoing counselling beyond the first 24 hours of birth [25, 26]. Within the WHO guidance for ongoing counselling, four conditions were mentioned (postpartum haemorrhage, pre-eclampsia/eclampsia, infection, and thrombo-embolism), and all except thromboembolism have been considered within the list of signs and symptoms. Thromboembolism was considered but disregarded by the Expert Committee as they were reported to be unlikely to occur in the first 24 hours after birth. From the three included categories, all signs and symptoms aligned with those described in the WHO signs and symptoms except epigastric abdominal pain. In the Delphi Survey only 66% of participants ranked this symptom category as critically important and as such it was excluded during sensitivity analysis.

Despite the risk of maternal mortality and morbidity, after caesarean birth, being five times higher than following vaginal birth, there were no CPGs for assessing signs and symptoms following caesarean birth [27–29]. Only one sign/symptom mentioned caesarean birth, and this was blood loss greater than 1000mls for postpartum haemorrhage. Interestingly, experts in postnatal care highlighted the need for inclusion of signs and symptoms specific to caesarean birth, both during discussions with the Expert Committee and during the Delphi Survey. Given the higher risks of morbidity and mortality associated with caesarean section, there is a need for specific guidance on the assessment of signs and symptoms following caesarean births. This should be separate to that for vaginal birth.

When preparing for the Delphi Surveys, there were often multiple ways to describe each sign and symptom based on differing country or setting. The need for careful attention of the language and phrasings used in a recommendation document is highlighted within the WHO handbook for guideline development [30]. Literature has reported on the pitfalls occurring particularly with patient reported tools, where poor language choices can lead to misinterpretation of signs and symptoms [31]. It is therefore imperative that beyond securing the signs and symptoms, attention is taken to ensure the phrasing and language used for the signs and symptoms are context specific to each setting.

## Conclusion

International expert consensus was achieved on a list of 19 key signs and symptoms spanning six condition categories that are important and deemed possible that mothers supported by their birth companions could assess in the first 24 hours postnatally. Further work is needed to ensure that this proposed list is adapted to individual regional and country settings to meet the needs of the women and birth companions in such settings in the context of self-monitoring in the immediate postnatal period.

## Figures and Tables

**Figure:**
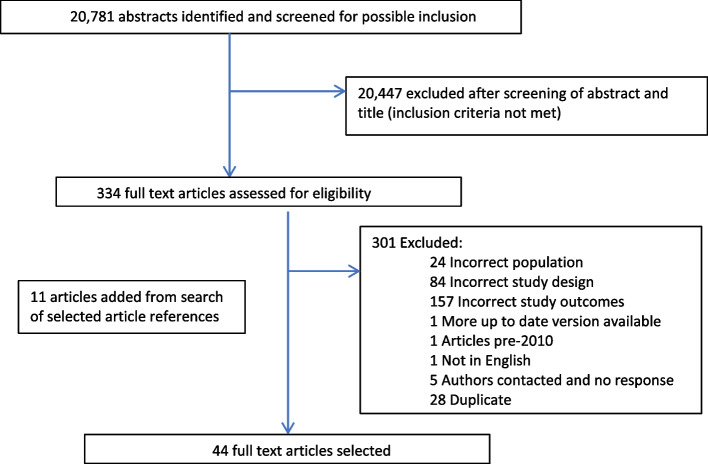
PRISMA flow chart of searches done

## Data Availability

No datasets were generated or analysed during the current study.

## Appendix 1

### Search Strategy

Search Matrix

1. Guideline

2. Guidelin*

3. SoP*

4. Standard operating procedur*

5. Tool

6. Tool*

7. Toolkit

8. Tool-kit

9. Tool-kit*

10. Toolkit*

11. Checklist

12. Check list

13. Check list*

14. Checklist*

15. Standard

16. Standard*

17. Protocol

18. Protocol*

19. Policy

20. Polic*

21. Position Statement*

22. Job Aid*

23. Consensus*

24. Recommendation

25. Recommendation*

26. Screening

27. Assess

28. Assessment

29. Assessment*

30. Report*

31. Criteria

32. Criteri*

33. Care-plan

34. Careplan

35. Care plan

36. Process*

37. Postnatal

38. Post-natal

39. Post natal

40. Postpartum

41. Post partum

42. Post-partum

43. Intrapartum

44. Intra partum

45. Intra-partum

46. Pregnanc*

47. Maternal

48. Maternity

49. Matern*

50. Afterbirth

51. After birth

52. After-birth

53. Postbirth

54. Post birth

55. Post-birth

56. 1 OR 2 OR 3 OR 4 OR 5 OR 6 OR 7 OR 8 OR 9 OR 10 OR 11 OR 12 OR 13 OR 14 OR 15 OR 16 OR 17 OR 18 OR 19 OR 20 OR 21 OR 22 OR 23 OR 24 OR 25OR 26 OR 27 OR 28 OR 29 OR 30 OR 31 OR 32 OR 33 OR 34 OR 35 OR 36 57. 37 OR 38 OR 39 OR 40 OR 41 OR 42 OR 43 OR 44 OR 46 OR 47 OR 48 OR 49 OR 50 OR 51 OR 51 OR 53 OR 54 OR 55 58. 56 AND 57

List of published databases searched

-PubMed – Medline,

-Excerpta Medica Database (EMBASE),

-Cochrane Library,

-Cumulative Index to Nursing and Allied Health Literature Cumulative Index to Nursing and Allied Health Literature (CINAHL),

-Latin-American and Caribbean System on Health Sciences Information (LILACS),

-Turning Research Into Practice (TRIP),

-Canadian Medical Association Infobase,

-E-guidelines,

-Geneva Foundation for Medical education and Research,

-WHO Guideline Repository,

-National guideline clearing house,

-International Guideline library,

-African Index Medicus,

-African Journals Online,

-Global Health Library

List of published databases searched

-Google Scholar,

-Scopus,

-Grey Matters

List of professional organisations searched

Obstetrics and midwifery societies

•Royal College of Obstetricians and Gynaecologists (RCOG)

•American College of Obstetricians and Gynaecologists (ACOG)

•The International Federation of Gynaecology and Obstetrics (FIGO)

•The Chinese Society of Obstetrics and Gynaecology (CSOG)

•National College of Gynaecologists and Obstetricians ‐ France (CNGOF)

•The International Society of Ultrasound in Obstetrics and Gynaecology (ISUOG)

•Japan Society of Obstetrics and Gynaecology (JSOG)

•Society of Obstetricians and Gynaecologists of Canada (SOGC)

•Royal Australian and New Zealand College of Obstetrician and Gynaecologists (RANZCOG)

•European Board & College of Obstetrics and Gynaecology (EBCOG)

•Royal College of Midwives

•International Confederation of Midwives

•The Global Library of Women’s medicine (GLOWM)

•The German Society for Gynaecology and Obstetrics (DGGG)

•Czech Gynaecological and Obstetrical society

•Nordic Societies of Obstetrics and Gynaecology

•Italian Society of Gynaecology and Obstetrics

•Association of the Hungarian Obstetricians and Gynaecologists

•Italian Association of hospital Obstetricians and Gynaecologists (AOGOI)

•Swedish Society of Obstetricians and Gynaecologists (SFOG)

•The neverlands society of obstetrics and gynaecology

•Nordic Federation of Obstetrics and Gynaecology (NFOG)

•Federation of Obstetric and Gynaecological Societies of India (FOGSI)

•European Network of Trainees in Obstetrics and Gynaecology (ENTOG)

•Russian Society of Obstetrics and Gynaecology (RSOG)

•Federation of Latin American Societies of Obstetrics and Gynaecology (FLAGSOG)

Clinical Practice and Guidelines Societies

•Guidelines International Network Library

•Academy of Medicine of Malaysia

•New Zealand and Australian Clinical Practice Guidelines (NZA)

•Australian Clinical Practice Guidelines

•Guidelines International Network

•Centre for Reviews and Dissemination

•Campbell Collaboration

•EQUATOR Network

National Health Organisations

•CDC

••ECRI

••Institute for Clinical Systems Improvement

••HAS- French National Authority for Health

••Belgium Healthcare Knowledge Centre

•Australian Government National Health and Medical Research Council (NHMRC)

•Canadian CPG Infobase

•The National Institute for Health and Care Excellence (NICE)

•Scottish Intercollegiate Guidelines Network (SIGN)

•Agency Healthcare Research and Quality (AHRQ)

•United States Agency for International Development (USAID)

•Institute for Clinical Systems Improvement (ICSI)

•Institute for healthcare improvement (IHI)

Multilateral Health Organisations

•World Health Organisation

•UNICEF

••UNFPA

Non-governmental key specialist organisations

•Johns Hopkins Program for International Education in Gynaecology and Obstetrics (JHPEIGO)

•Effective Public Health Practice Project

•Joanna Briggs Institute

•Women Deliver

## Appendix 2

List of Expert Committee members

**Table:**

**Name**	**Role**	**Affiliated Organisation**
Andrew Weeks	Professor	Sanyu Research Unit, University of Liverpool, United Kingdom
Tina Lavender	Professor	Liverpool School of Tropical Medicine, United Kingdom
Etienne Langlois	Scientist	Partnership for Maternal and Newborn Child Health (PMNCH), Switzerland
Matthews Mathai	Professor	Liverpool School of Tropical Medicine, United Kingdom
Angela Hancock	Consultant Midwife	Royal Stoke University Hospital, United Kingdom
Enid Mwebaza	Community Liason Officer	Sanyu Africa Research Institute, Uganda
Sam Ononge	Senior Lecturer	Makerere University, Uganda
Lenka Benova	Professor	The Institute of Tropical Medicine (ITM) in Antwerp, Belgium
Elizabeth Ayebare	Senior Lecturer	Makerere University, Uganda

## Appendix 4

List of demographics of included guidelines

**Table:**

Title	Year Published	Commissioning Agency	Stage in Pregnancy (Antepartum, Intrapartum, Postpartum)	Proposed Setting for use
Quantitative Blood Loss in Obstetric Haemorrhage: ACOG COMMITTEE OPINION, Number 794. [32]	2019	ACOG	P	USA
ACOG Practice Bulletin No. 212: Pregnancy and Heart Disease. [33]	2019	ACOG	A, I, P	USA
ACOG Practice Bulletin No. 196: Thromboembolism in Pregnancy. [34]	2018	ACOG	A, I, P	USA
Emergent Therapy for Acute-Onset, Severe Hypertension During Pregnancy and the Postpartum Period. Committee Opinion, Number 692. [35]	2017	ACOG	A, I, P	USA
Hypertension in Pregnancy. Report of the American College of Obstetricians and Gynaecologists’ Task Force on Hypertension in Pregnancy. [36]	2013	ACOG	A, I, P	USA
ACOG Practice Bulletin No. 183: Postpartum Hemorrhage. [37]	2017	ACOG	P	USA
Guidelines of the American Thyroid Association for the diagnosis and management of thyroid disease during pregnancy and postpartum. [38]	2011	American Thyroid Association	A, I, P	USA
UK guidelines on the management of iron deficiency in pregnancy. [39]	2019	British Society of Haematologists	A, I, P	UK
Prevention and treatment of postpartum haemorrhage in low-resource settings. [40]	2012	FIGO	A, I, P	Global
FOGSI’s GCPR on Hypertensive Disorders in Pregnancy (HDP) 2019. [41]	2019	Federation of Obstetric and Gynaecological Societies of India (FOGSI)	A, I, P	India
FOGSI good clinical practice recommendation on management of Iron deficiency Anaemia in pregnancy. [42]	2017	FOGSI	A, I, P	India
Peripartum Haemorrhage, Diagnosis and Therapy. Guideline. [43]	2018	German Society of Gynaecology and Obstetrics (DGGG), the Austrian Society of Gynaecology and Obstetrics (OEGGG) and the Swiss Society of Gynaecology and Obstetrics (SGGG)	P	Germany, Austria, Switzerland
Clinical practice guideline the diagnosis and management of severe pre-eclampsia and eclampsia. [44]	2011	Institute of Obstetricians and Gynaecologists, Royal College of Physicians of Ireland	A, I, P	Ireland
Clinical practice guideline management of urinary retention in pregnancy, post-partum and after gynaecological surgery. [45]	2018	Institute of Obstetricians and Gynaecologists, Royal College of Physicians of Ireland	A, I, P	Ireland
Clinical practice guideline management of urinary tract infections in pregnancy. [46]	2018	Institute of Obstetricians and Gynaecologists, Royal College of Physicians of Ireland	A, I, P	Ireland
The classification, diagnosis and management of the hypertensive disorders of pregnancy: A revised statement from the ISSHP. [47]	2014	International Society for the Study of Hypertension in Pregnancy	A, I, P	Global
Guidelines for obstetrical practice in Japan: Japan Society of Obstetrics and Gynaecology (JSOG) and Japan Association of Obstetricians and Gynaecologists (JAOG). [48]	2014	JSOG/JAOG	A, I, P	Japan
Clinical Practice Guidelines for prevention, detection early and treatment of complications of pregnancy, childbirth, or the puerperium. [49]	2013	Ministry of Health Columbia	A, I, P	Columbia
National Standards for maternal and newborn care. [50]	2010	Ministry of Health Honduras	A, I, P	Honduras
Patient blood management in obstetrics: prevention and treatment of postpartum haemorrhage. A NATA consensus statement. [51]	2019	NATA/FIGO/IFG/EBCOG/ESA	P	Europe
Patient blood management in obstetrics: management of anaemia and haematinic deficiencies in pregnancy and in the post‐partum period: NATA consensus statement. [52]	2017	NATA/FIGO/IFG/EBCOG/ESA	A, I, P	Europe
S3-Guidelines for the Treatment of Inflammatory Breast Disease during the Lactation Period. [53]	2013	National Breastfeeding Committee and German Society for Gynaecology and Obstetrics	P	Germany
Observation of Mother and Baby in the Immediate Postnatal Period: Consensus statements guiding practice. [54]	2012	New Zealand Ministry of Health	P	New Zealand
Antenatal and postnatal mental health: clinical management and service guidance. [55]	2014	NICE	A,P	UK
Postnatal care up to 8 weeks after birth. [56]	2015	NICE	P	UK
Hypertension in pregnancy (NICE clinical guideline 107). [57]	2019	NICE	A, I, P	UK
Mental Health Care in the Perinatal Period. [58]	2018	RANZCOG	P	Australia + New Zealand
Management of Women with Mental Health Issues during Pregnancy and the Postnatal Period. [59]	2011	RCOG	A, I, P	UK
Sepsis following Pregnancy, Bacterial (Green-top Guideline No. 64b). [60]	2012	RCOG	P	UK
Prevention and Management of Postpartum Haemorrhage. [61]	2016	RCOG	P	UK
Thromboembolic Disease in Pregnancy and the Puerperium: Acute Management. [62]	2015	RCOG	A, I, P	UK
Management of perinatal mood disorders. [63]	2012	SIGN	A, I, P	Scotland
SOGP Recommendation for the Diagnosis and Management of Iron Deficiency Anaemia in Pregnancy and Postpartum. [64]	2018	SOGP (Society of Obstetricians & Gynaecologists of Pakistan)	A, I, P	Pakistan
SOMANZ guidelines for the investigation and management sepsis in pregnancy. [65]	2017	SOMANZ	A, I, P	Australia + New Zealand
Guideline for the Management of Hypertensive Disorders of Pregnancy. [66]	2014	SOMANZ	A, I, P	Australia + New Zealand
Recommendations for the diagnosis and treatment of deep venous thrombosis and pulmonary embolism in pregnancy and the postpartum period. [67]	2011	SOMANZ/ ASTH	A, I, P	Australia + New Zealand
Guidelines for Hypertension in Pregnancy. [68]	2019	Swedish Society of Obstetricians and Gynaecologists (SFOG)	A, I, P	Sweden
Provision of care, treatment and services standards for maternal safety. [69]	2013	The Joint Commission	A, I, P	USA
Ugandan Clinical Guidelines 2016	2016	Ugandan National Council for Science and Technology	A,I,P	Uganda
Consensus-derived clinical decision rules to guide advanced imaging decisions for pulmonary embolism in pregnancy and the postpartum period. [70]	2018	University of Sheffield	A, I, P	U.K.
WHO Pregnancy, childbirth, postpartum and newborn care A guide for essential practice (3rd edition). [71]	2015	WHO	A, I, P	Global
WHO recommendations on postnatal care of the mother and newborn. [72]	2013	WHO	P	Global
WHO recommendations on maternal health: guidelines approved by the WHO Guidelines Review Committee. [73]	2017	WHO	A, I, P	Global
Managing Complications in Pregnancy and Childbirth: A Guide for Midwives and Doctors. 2nd ed. [74]	2017	WHO	A, I, P	Global

## Appendix 5

List of signs and symptoms to be reviewed by first round of Delphi Survey

**Table:**

**Condition**	**Sign/Symptom**
**Postpartum Haemorrhage**	Blood loss of 2 cups after a vaginal birth Blood loss more than 1 pad or cloth soaked in <5 minutes Increase in blood loss rather than decrease after birth Constant trickling of blood Blood loss >2-3 pads soaked in 20-30 minutes after birth Intermittent heavy bleeding Passing a clot the size of or bigger than an egg Soaking through 1 pad per hour Palpitations Cool Temperature of skin Cold extremities Moist skin Clammy skin Change in Consciousness Altered mental state Anxiousness Confusion Faint Dizzy Pallor Pallor of palms Fast respiratory rate Uterine tenderness Weak pulse Sweating Nausea Thirsty Fast Heart Rate Soft Slack Uterus Not passed Urine for 6 hours
**Genital Tract Sepsis**	Fever Offensive vaginal loss Offensive odour of perineum Foul smelling vaginal discharge Bad smelling vaginal blood or discharge Abdominal pain Uterine tenderness Uncontrollable shivering Chills Rigors Hypothermia Perineal pain Stinging Perineal inflammation Heart racing Fast laboured breathing Lethargy Very weak/ cannot stand Confusion Mild agitation Altered Mental state Vomiting Diarrhoea Anxiety/ impending doom enlargement of wound sudden opening up of wound Sudden Leakage of blood
**Cardiovascular Disease**	Shortness of breath Trouble breathing Swelling of legs Palpitations Fatigue Overwhelming tiredness Chest pain Cough Syncope Fast Heart Rate Fast breathing
**Pre-eclampsia**	Visual disturbance Blurred vision Problems or changes with vision Flashing before the eyes Persistent visual impairment Blindness Severe headache Persistent, new headache Worsening and increasing in frequency headache Headache not alleviated by analgesics or medicine Right upper quadrant pain Severe pain below the ribs Upper right Belly pain Nausea Vomiting Sudden swelling of face, hands or feet Convulsions Seizures Altered consciousness Altered mental state Shortness of breath Chest pain Feeling faint Dizziness Giddiness Tea/coffee coloured urine Not PU for 6 hours
**Urinary dysfunction**	Leakage of urine Urinary incontinence Urine dribbling Dysuria Incomplete emptying Unable to urinate easily Bladder Pain Bladder tenderness Increased urinary frequency Low grade fever Pyrexia Not passing Urine within 6 hours of birth Voiding small amounts Slow intermittent stream Haematuria Polyuria Straining to void
**Anaemia**	Pallor Severe pallor Generalised weakness Easy tiredness Extreme tiredness Palpitations Rapid heartbeat Shortness of breath Difficulty breathing Headache Frontal headache Dizziness
**Postpartum Psychosis**	Sleep Disturbance Unable to sleep Feeling of no need for sleep Obsessive thoughts about the baby Severe depression Despair Anxiety Suicidal/ Infanticidal impulses Believing strange things that can’t be true (delusions) Hearing, seeing or feeling things that are not there (hallucinations)
